# Assessing Food Safety Risks in Homemade Fermented Beverages: A Case Study with Quinoa Rejuvelac

**DOI:** 10.3390/life16040556

**Published:** 2026-03-28

**Authors:** Cristiana Guimarães Brasileiro, Marcos Thalyson da Conceicao Moreno, Eidy de Oliveira Santos, P. Saranraj, Alexander Machado Cardoso, Jessica Manya Bittencourt Dias Vieira

**Affiliations:** 1 Department of Biology, Rio de Janeiro State University (UERJ), Rio de Janeiro 20550-013, RJ, Brazil; cristianaguimaraes19@gmail.com (C.G.B.); marcosthalyson250394@gmail.com (M.T.d.C.M.); eidyos@gmail.com (E.d.O.S.); 2 PG and Research Department of Microbiology, Sacred Heart College (Autonomous), Tirupattur 635 601, Tamil Nadu, India; microsaranraj@gmail.com

**Keywords:** artisanal fermented beverages, spontaneous fermentation, food safety, seed-borne endophytes

## Abstract

Spontaneous fermentation processes can promote uncontrolled microbial growth and increase the risk of foodborne contamination, making the characterization of artisanal beverages essential for consumer safety. This study investigated the microbial composition of quinoa-based rejuvelac, a homemade fermented drink often perceived as a functional food, with the objective of identifying potential microbiological hazards associated with its preparation. High-throughput sequencing of the 16S rRNA V3–V4 region was combined with shotgun metagenomics to profile bacterial communities and recover metagenome-assembled genomes. The analysis revealed a strong dominance of *Pseudomonadales*, mainly *Pseudomonas*, *Acinetobacter*, *Enterobacter* and *Burkholderiales*, while lactic acid bacteria typically responsible for stable and safe fermentations were not detected. Shotgun metagenomics recovered medium- to high-quality genomes from *Burkholderiaceae* and *Clostridiales*, supporting the overrepresentation of non-beneficial taxa and indicating deviations from expected fermentation microbiota. These results show that the spontaneous preparation of rejuvelac may favor bacterial groups associated with environmental contamination rather than fermentative pathways, underscoring the importance of hygiene practices, controlled starter cultures and monitoring strategies to mitigate microbiological risk. The study highlights the need for improved safety standards in artisanal fermented foods to prevent unintended microbial contamination and protect consumers.

## 1. Introduction

The growing demand for health-promoting and sustainable dietary alternatives has significantly increased the consumption of artisanal fermented foods, which are often considered as functional due to their potential probiotic properties and benefits to gut health [1]. Among these preparations, rejuvelac stands out as an enzymatic beverage traditionally obtained through the spontaneous fermentation of sprouted grains, such as wheat, rye, or, more recently, pseudocereals like quinoa (*Chenopodium quinoa* Willd.) [2,3]. Quinoa is highly valued for its superior nutritional profile, containing high-biological-value proteins, dietary fibers, and bioactive compounds, making it a promising substrate for plant-based fermented beverages [4].

However, the domestic production of rejuvelac relies predominantly on spontaneous fermentation, a process governed by the natural microbiota present on the grains and within the preparation environment. While fermentation is an ancestral preservation method, the absence of standardized starter cultures and the limited control over physicochemical parameters, such as pH and temperature, pose significant food safety challenges [5]. Under inadequate conditions, the fermentative pathway may be diverted, allowing the proliferation of spoilage or pathogenic microorganisms instead of favoring the growth of beneficial lactic acid bacteria (LAB), such as *Lactobacillus* spp., which are responsible for product acidification and microbiological safety [6].

A critical and often overlooked factor in the spontaneous fermentation of quinoa is the role of seed-borne endophytic microorganisms. Quinoa seeds harbor a complex and stable endophytic community, including genera such as *Bacillus*, *Pseudomonas*, and *Enterobacter*, which contribute to plant fitness and stress resistance [7,8]. During the soaking and sprouting stages of rejuvelac preparation, these endophytes can be released into the aqueous medium, potentially dominating the early stages of fermentation. If the environment does not favor rapid acidification by LAB, these endogenous taxes, some of which are associated with environmental contamination or opportunistic pathogenicity, may persist or even thrive, compromising the safety of the final beverage [9].

Recent studies have highlighted the microbiological risks associated with uncontrolled fermented beverages, where the presence of opportunistic taxa and environmental contaminants can endanger consumer health [10]. Detailed characterization of the microbiota in these beverages is therefore essential to identify potential hazards and establish guidelines for safe artisanal manufacturing. With the advent of high-throughput sequencing technologies, it is now possible to explore microbial diversity with high resolution, overcoming the limitations of traditional culture-dependent methods [11]. In this context, the present study aimed to investigate the microbial composition of artisanal prepared quinoa rejuvelac using a combined approach of 16S rRNA gene sequencing (V3–V4 region) and shotgun metagenomics. By recovering metagenome-assembled genomes (MAGs), we sought to identify the primary bacterial groups present and evaluate the occurrence of taxa associated with food safety risks. Our results reveal an unexpected dominance of environmental and potentially pathogenic groups, underscoring the critical need for monitoring and standardization in home-based fermented food production.

## 2. Materials and Methods

### 2.1. Preparation of Quinoa Rejuvelac Samples

**Figure 1:**
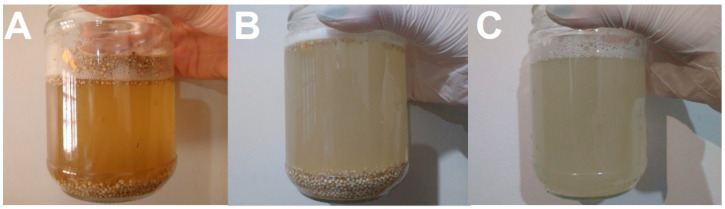
Preparation steps of rejuvelac. (**A**) Quinoa undergoing germination during the first 24 h; (**B**) Fermentation process, 48 h after water replacement; (**C**) Finished rejuvelac, with the quinoa grains removed from the liquid.

Quinoa rejuvelac was prepared following traditional artisanal protocols adapted for laboratory conditions. Quinoa (*Chenopodium quinoa* Willd) seeds were surface sterilized using a standard disinfection protocol to eliminate external contaminants. The seeds were initially agitated for 30 min in a solution containing 100 mL of distilled water supplemented with 10 µL of Tween 20 to facilitate the removal of surface impurities. Excess foam was then removed by rinsing the grains with sterile water. Subsequently, the quinoa seeds were transferred to a laminar flow hood and immersed in 70% ethanol for 10 min. Following ethanol treatment, the grains were rinsed three times with sterile water to eliminate residual ethanol. The seeds were then subjected to surface sterilization using a solution of commercial bleach (2.5% sodium hypochlorite) diluted to 20% (*v*/*v*) in distilled water. After 15 min of exposure, the grains were thoroughly rinsed three times with sterile water to ensure complete removal of the sterilizing agent. Germination assays were conducted in sterile glass jars. Approximately 40 g of white quinoa grains were submerged in 500 mL of potable water over a 24 h period. After this time, the soaking water was discarded, and the grains were briefly rinsed with sterile potable tap water. A new volume of 500 mL of sterilized potable tap water was then added to support fermentation, resulting in the production of the beverage. The grains remained submerged for 48 h at room temperature (25 ± 2 °C), protected from light and excessive heat. At the end of the process, a turbid liquid with a slightly acidic odor was obtained, consistent with a decline in pH from 6.1 to 4.9 (Figure 1). Samples were collected in triplicate (three independent fermentations) for genomic DNA extraction.

### 2.2. DNA Extraction

Total microbial community DNA was extracted from the rejuvelac samples using the DNeasy^®^ PowerWater^®^ Kit (Qiagen, Hilden, Germany). For this procedure, approximately 100 mL of rejuvelac was vacuum-filtered through a 0.22 µm pore-size membrane filter. The extracted DNA was quantified using a NanoDrop^®^ 2000 spectrophotometer (Thermo Scientific, Waltham, MA, USA). One microliter of each sample was analyzed in duplicate to determine the DNA concentration (ng/µL). During quantification, the degree of purity (A260) and purity ratios were evaluated, including the DNA-to-protein ratio (260/280 ratio) and the ratio of DNA to other contaminants (260/230 ratio). Subsequently, the quality and integrity of the extracted DNA were confirmed by 1% (*w*/*v*) agarose gel electrophoresis. Once the suitability of the genetic material was confirmed, the samples were proceeded for high-throughput sequencing.

### 2.3. High-Throughput Sequencing and Bioinformatic Processing

For amplicon analysis, the V3–V4 hypervariable region of the 16S rRNA gene was amplified using universal primers 341F (5′-CCTACGGGNGGCWGCAG-3′) and 805R (5′-GACTACHVGGGTATCTAATCC-3′). Libraries were prepared following the standard Illumina protocol and sequenced on the MiSeq platform (Illumina, San Diego, CA, EUA) with paired-end chemistry (2 × 300 bp). For shotgun metagenomics, genomic DNA libraries were constructed using the Nextera XT DNA Library Prep Kit and sequenced on the HiSeq 4000 platform (Illumina) to obtain high depth reads. The processing of raw 16S rRNA sequences was performed using an integrated approach combining DADA2 and MOTHUR (v.1.48.0). Initially, the DADA2 package (v.1.16) in the R environment was used for quality filtering, sequencing error correction, and inference of Amplicon Sequence Variants (ASVs). The filtered and dereplicated sequences were then imported into MOTHUR for alignment against the SILVA database (v.138.2), taxonomic assignment and chimera removal via the VSEARCH algorithm.

### 2.4. Shotgun Metagenomic Analysis and Genome Reconstruction

Raw shotgun metagenomic reads were processed on the KBase platform (DOE Systems Biology Knowledgebase). Initial quality control was performed with FastQC, and adapter removal was conducted using Trimmomatic. *De novo* metagenome assembly was executed using the metaSPAdes assembler (v.3.15.3). For the recovery of metagenome-assembled genomes (MAGs), the assembled contigs were subjected to binning using the MaxBin2 and MetaBAT2 algorithms integrated within KBase. The quality of the recovered MAGs was assessed using CheckM, considering criteria of completeness (>70%) and contamination (<5%) for classification as medium- to high-quality genomes. Taxonomic classification of the MAGs was performed via GTDB-Tk (Genome Taxonomy Database Toolkit) and TYGS (Type Strain Genome Server). Functional annotation and metabolic pathway identification were conducted using Prokka and KEGG (GhostKOALA), with a focus on detecting genes associated with pathogenicity and antibiotic resistance.

### 2.5. Statistical Analysis

Significant differences in the relative abundance of taxa among replicates were evaluated by analysis of variance (ANOVA) followed by Tukey’s test (*p* < 0.05). All graphical visualizations were generated using the GraphPad Prism (v.8.0.2).

## 3. Results

### 3.1. Microbial Community Composition by 16S rRNA Gene Sequencing

**Figure 2:**
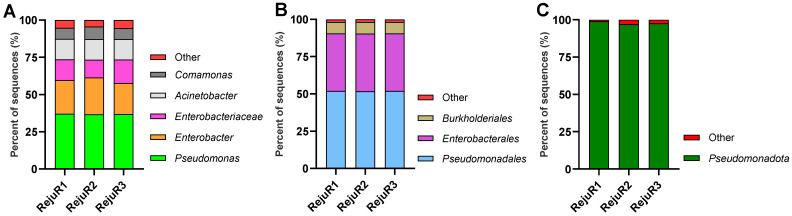
Taxonomic composition of the bacterial community in quinoa rejuvelac samples. (**A**) Relative abundance of dominant bacterial genera identified across the three samples (RejuR1–RejuR3). (**B**) Relative abundance of the main bacterial orders and (**C**) phyla detected in the same samples. Bars represent the percentage of sequences assigned to each taxonomic group, with minor taxa (<2%) grouped as Other.

High-throughput sequencing of the 16S rRNA V3–V4 region revealed a distinct microbial community profile in the spontaneously fermented quinoa rejuvelac samples. Contrary to expectations for a beneficial fermented beverage, the analysis showed a strong and consistent dominance of *Pseudomonadota* (98.97–97.59%) accounting for most observed sequences across all replicates. Specifically, the most abundant genera identified within this phylum included *Pseudomonas*, *Enterobacter*, and *Acinetobacter* alongside a significant presence of taxa belonging to the order *Pseudomonadales*, *Enterobacterales*, and *Burkholderiales* (Figure 2). Notably, lactic acid bacteria, typically associated with stable and safe fermentations and responsible for acidification, were either absent or detected at negligible levels, failing to establish a dominant presence in the rejuvelac microbiota. This absence suggests a deviation from a typical beneficial fermentation process.

### 3.2. Metagenome-Assembled Genomes and Taxonomic Insights

Shotgun metagenomics further supported the 16S rRNA gene sequencing findings, providing deeper taxonomic and genomic insights into the microbial community. From the metagenomic data, several medium- to high-quality metagenome-assembled genomes (MAGs) were successfully recovered (Table 1).

Shotgun metagenomic assembly and binning resulted in the recovery of eleven genomic bins representing bacterial taxa associated with the quinoa rejuvelac microbiota. Quality assessment using single-copy marker genes revealed variable levels of genome completeness and contamination across bins. Among the recovered bins, three exhibited high completeness values (>85%), including bins affiliated with *Burkholderiaceae* and *Clostridiales*, with estimated completeness 95.83%, and 98.39%, respectively, and low contamination levels (<3%). An additional bin assigned to *Sphingomonadales* showed moderate completeness (72.24%) with minimal contamination (0.68%). Other bins displayed lower completeness values (<60%) and were broadly classified at higher taxonomic levels such as Bacteria or *Enterobacteriaceae* (Table 1). Overall, these results indicate that members of *Burkholderiales*, *Burkholderiaceae*, and *Clostridiales* represent some of the most genomically well-resolved and potentially abundant taxa within the reconstructed metagenomic dataset, supporting the taxonomic trends observed in the 16S rRNA gene sequencing analysis. The genomic information from these MAGs corroborated the overrepresentation of non-beneficial taxa, reinforcing the observation that the spontaneous fermentation process favored microorganisms often associated with environmental niches rather than typical food fermentation pathways. Functional annotation of these MAGs indicated the presence of genes related to environmental adaptation and potential opportunistic characteristics, further highlighting the microbial composition’s divergence from a desirable profile for a functional food product (Supplementary Tables S1 and S2).

High-resolution taxonomic identification revealed that the most complete metagenome-assembled genomes (MAGs) recovered from quinoa rejuvelac are closely related to known opportunistic pathogens. Bin 4 was identified as a member of the genus *Ralstonia*, exhibiting the highest digital DNA-DNA hybridization (dDDH d0) value of 74.7% with *Ralstonia pickettii* NBRC 102503 (Supplementary Table S3). This value exceeds the 70% threshold for species delimitation, confirming its identification as *R. pickettii*. In contrast, Bin 5 was phylogenetically placed within the order *Clostridiales*, showing the highest proximity to *Clostridium sartagoforme* JCM 1413 (dDDH d4 = 22.1%) and *Clostridium tertium* DSM 2485 (dDDH d4 = 22.0%). Although the dDDH values for Bin 5 remain below the species-level threshold, its close affiliation with these *Clostridium* species, which are recognized opportunistic pathogens, underscores the potential microbiological risks associated with the beverage (Supplementary Table S4).

**Figure 3:**
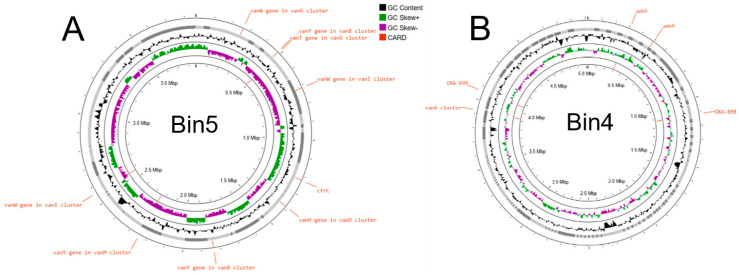
Circular genome maps of representative metagenome-assembled genomes (MAGs) Bin5 (**A**) and Bin4 (**B**) recovered from the quinoa rejuvelac metagenome, generated using the software Proksee. Concentric rings represent genomic features including GC content, GC skew, coding sequences, and predicted functional genes. Genes associated with antibiotic resistance were annotated based on comparisons with the Comprehensive Antibiotic Resistance Database (CARD) are highlighted in orange and mapped to their genomic positions.

Circular genome visualization of the reconstructed metagenome-assembled genomes revealed the genomic architecture and functional gene distribution of two of the most complete bins recovered from the quinoa rejuvelac metagenome (Figure 3). The maps generated showed well-defined genomic structures with conserved GC content and GC skew patterns across both genomes, supporting the integrity of the assemblies. Functional annotation identified multiple loci associated with antimicrobial resistance when screened against the Comprehensive Antibiotic Resistance Database (CARD).

### 3.3. Virulence Factors and Antimicrobial Resistance Profiling

Functional analysis of the most complete MAGs revealed a concerning repertoire of genes associated with pathogenicity and antibiotic resistance. Screening against the Virulence Factor Database (VFDB) identified multiple virulence determinants (Supplementary Tables S1 and S2). For Bin 4 (*Burkholderiaceae*), determinants across several categories were identified, including adherence (Type IV pili: *pilB*, *pilD*), antiphagocytosis (Capsule I: *manC*, *wcbA*, *wcbB*, *wcbC*, *wcbD*, *wcbP*, *wcbQ*, *wcbR*, *wcbS*, *wcbT*), and motility (Flagella: *flhC*, *flhD*, *fliQ*, *motB*). Additionally, genes related to iron uptake (*ccmF*), serum resistance (*brkB*), and endotoxin production (*kdsA*, *kdsB*) were detected. For Bin 5 (*Clostridiales*), the VFDB analysis revealed genes associated with adherence and colonization, such as the fibronectin-binding protein (*fbpA/fbp68*), the chaperone groEL, and the CD0873 adhesin. Genes involved in immune evasion and antiphagocytosis were also present, including capsule-related genes (*cps2T*, *epsE*, *gtaB*). Furthermore, Bin 5 harbored genes for hemolysins, iron and copper uptake (*cbrD*, *vctC*, *ctpV)*, and components of a Type III secretion system (*cdsN*). Regulatory genes associated with virulence, such as *virR* and *virS*, were also identified.

Analysis of antibiotic resistance genes within the reconstructed metagenome-assembled genomes revealed the presence of multiple antimicrobial resistance determinants, several genes belonging to the vancomycin resistance (*van*) gene clusters were detected, including *vanW*, *vanY*, *vanT*, and *vanH*, which are typically associated with resistance to glycopeptide antibiotics such as vancomycin and teicoplanin. These genes act primarily through antibiotic target alteration, modifying the peptidoglycan precursors and reducing antibiotic binding. In addition, the *cfrC* gene, encoding a 23S rRNA methyltransferase, was identified and is known to confer resistance to multiple antibiotic classes including lincosamides, streptogramins, oxazolidinones, and phenicols. Resistance determinants associated with chloramphenicol and florfenicol were also detected. In Bin4, resistance genes related to multiple antibiotic classes were also identified. These included the *adeF* gene, a component of the resistance-nodulation-cell division (RND) efflux pump system, which contributes to resistance against fluoroquinolones and tetracyclines by actively exporting antibiotics from the cell. Additionally, β-lactam resistance genes such as *OXA*_899_, encoding an *OXA*-type β-lactamase, were detected and are associated with resistance to penicillins and cephalosporins. Like Bin5, genes belonging to the *vanA*-type glycopeptide resistance cluster were also identified. Together, these findings indicate that the reconstructed genomes harbor a diverse resistome, including genes mediating target modification, antibiotic inactivation, and multidrug efflux, suggesting that members of the microbial community present in the rejuvelac fermentation environment may act as reservoirs of clinically relevant antibiotic resistance genes.

## 4. Discussion

Recent studies have demonstrated that quinoa seeds and rejuvelac produced from their sprouted grains harbor distinct microbial profiles. Quinoa seeds harbor a complex and stable endophytic community, including genera such as *Bacillus*, *Pseudomonas*, and *Enterobacter* [7,8]. In contrast, rejuvelac obtained under appropriate fermentation conditions is typically characterized by the predominance of lactic acid bacteria (LAB), including genera such as *Pediococcus* and *Weissella* [2], reflecting a microbial shift driven by fermentation dynamics and acidification.

Traditional and safe fermentations are typically characterized by the rapid proliferation and metabolic activity of LAB, which produce lactic acid and other antimicrobial compounds, leading to a swift drop in pH. This acidification creates an environment inhospitable to many spoilage and pathogenic microorganisms, thereby ensuring product stability and safety [12]. The lack of LAB dominance in our rejuvelac samples suggests that this crucial protective mechanism was not effectively established. Instead, the observed microbial community reflects a selection for environmental bacteria, many of which are known to be opportunistic pathogens or spoilage agents [13]. For instance, *Pseudomonas* spp. are common environmental bacteria often associated with food spoilage, while *Acinetobacter* and *Enterobacter* species can be opportunistic human pathogens [14,15]. The presence of *Burkholderiales*, further confirmed by the recovery of MAGs from *Burkholderiaceae*, is particularly concerning, as some members of this order are known plant and human pathogens [16].

The microbial landscape of spontaneously fermented quinoa rejuvelac, as revealed by both 16S rRNA gene sequencing and shotgun metagenomics, presents a significant departure from the expected profile of a safe and beneficial fermented food. The overwhelming dominance of *Pseudomonadota*, particularly genera such as *Pseudomonas*, *Acinetobacter*, *Enterobacter*, and members of *Burkholderiales*, coupled with the conspicuous absence of lactic acid bacteria (LAB), raises critical concerns regarding the food safety of this artisanal beverage. Our findings strongly support the hypothesis that the seed-borne endophytic microbiota of quinoa plays a pivotal role in shaping the microbial community during spontaneous fermentation, especially in the absence of controlled starter cultures. Quinoa seeds harbor diverse endophytes, including *Pseudomonas* and *Enterobacter* species [7,8]. The initial sanitization step with hypochlorite, while reducing surface contaminants, may not entirely eliminate these internal seed-borne microorganisms. During the soaking and germination phases, these endophytes are released and, in the absence of competitive LAB, can readily colonize the fermentation medium. This scenario contrasts sharply with fermentations initiated by robust starter cultures, where the inoculated microorganisms rapidly outcompete indigenous flora, guiding the fermentation towards a desired and safe outcome [17].

Quinoa seeds are naturally rich in saponins, a class of amphipathic glycosides widely recognized for their antimicrobial activity [18]. These compounds can disrupt cell membranes, particularly in Gram-positive bacteria, which lack the protective outer membrane present in Gram-negative organisms [19]. This structural difference suggests that saponins may exert a selective inhibitory effect on lactic acid bacteria (LAB), while allowing more resistant Gram-negative taxa, such as members of *Pseudomonas*, *Enterobacter*, and *Burkholderiales*, to persist or even dominate during fermentation. In this context, the strong prevalence of *Pseudomonadota* observed in our samples may not only reflect the release of seed-borne endophytes during germination but also a selective ecological pressure imposed by residual saponins, which could hinder LAB establishment and delay acidification. This interpretation is supported by previous studies demonstrating that quinoa-associated endophytic communities are highly resilient and adapted to the plant’s chemical environment [8]. Therefore, saponins may play a dual role in rejuvelac fermentation: while contributing to the intrinsic antimicrobial defense of the seed, they may inadvertently bias the fermentation trajectory toward non-LAB-dominated communities, particularly under uncontrolled conditions.

The recovery of medium- to high-quality MAGs from *Burkholderiaceae* and *Clostridiales* further substantiates the presence and metabolic activity of non-beneficial taxa. While *Clostridiales* encompasses a broad range of bacteria, including some beneficial gut commensals, certain members are known for producing toxins or contributing to spoilage [20,21]. The overall microbial profile, characterized by a high abundance of *Pseudomonadota* and the absence of LAB, indicates that the spontaneous preparation of quinoa rejuvelac, under the conditions tested, inadvertently selected for bacterial groups associated with environmental contamination rather than fermentative pathways beneficial for food safety.

*Pseudomonadales* typically lack a complete, canonical Embden Meyerhof (EMP) glycolysis and generally do not employ the heterolactic phosphoketolase (PK) pathway; instead, they rely on the Entner–Doudoroff (ED) pathway and a versatile array of catabolic routes for complex organic matter [22]. Many *Pseudomonadales* species either lack key upper glycolysis enzymes (notably phosphofructokinase) or exhibit such low EMP pathway flux that glucose dissimilation proceeds mainly via oxidation and the ED route rather than classic glycolysis [23]. Under aerobic conditions, glucose is often oxidized through the periplasmic gluconate and 2 keto gluconate branch, feeding into the ED pathway to yield pyruvate with minimal fermentation type acid production, explaining the absence of glycolysis phenotype in classic physiological descriptions [24]. The phosphoketolase (PK) pathway is characteristic of certain lactic acid bacteria and some cyanobacteria, but it is not a dominant energy yielding route in *Pseudomonadales*; genomic and metabolic data show that these *Pseudomonadota* do not rely on PK driven sugar splitting for central carbon metabolism. Instead, *Pseudomonadales* use ED, pentose phosphate, and TCA cycle linked fluxes for sugar and organic acid utilization, so the absence of a PK pathway is functionally compensated by these alternative routes [25]. *Pseudomonadales* are renowned for their broad substrate spectrum, encoding numerous inducible catabolic operons for aromatic hydrocarbons, organic acids, amino acids, and other complex organics rather than long chain polymers [26]. Species related to *Pseudomonadales* employ diverse oxygenase-based systems, as well as specialized pathways for amino acid, nucleotide derived, and xenobiotic moieties, enabling them to thrive on mixed organic matter mixtures in soils, water, and wastewater [27].

The identification of *Ralstonia pickettii* and a lineage closely related to *Clostridium tertium* and *C. sartagoforme* in quinoa rejuvelac provides a clear genomic basis for the food safety concerns raised by this study. *R. pickettii* is a well-documented opportunistic pathogen often associated with healthcare-related infections due to its ability to survive in nutrient-poor environments and resist various disinfectants [28]. Its presence in a homemade beverage, likely to originate from the seed-borne endophytic community or environmental water sources, is particularly alarming given its potential to cause bacteremia and respiratory infections in vulnerable individuals [29]. Similarly, the detection of *Clostridium* species like *C. tertium* is significant; although traditionally considered a low-virulence commensal, it has emerged as a cause of serious infections, including neutropenic enterocolitis and sepsis, particularly in immunocompromised hosts [30]. The co-occurrence of these taxa, combined with the diverse repertoire of virulence factors and antimicrobial resistance genes identified in their genomes, suggests that the spontaneous fermentation of quinoa rejuvelac may inadvertently create a selective environment for pathogens that are resilient to standard hygiene practices.

The germination process provides favorable conditions for microbial proliferation, including potential bacterial pathogens. The presence of *Salmonella* spp., *Enterobacter* spp., and *Listeria* spp. has been previously reported in homemade wheat rejuvelac, whereas no pathogens were detected in sterilized preparations or in samples inoculated with lactic acid bacteria [31]. These observations are consistent with our findings, in which spontaneous fermentation did not result in the establishment of a LAB-dominated community, but instead favored the proliferation of environmental and opportunistic taxa, particularly within *Pseudomonadota*. In contrast, several studies have demonstrated that quinoa-based fermentations can successfully develop LAB-dominated microbiota under appropriate conditions. Controlled or semi-controlled fermentations have reported rapid acidification, with pH values decreasing to 3.8–4.5 within 48 h, alongside high viable LAB counts. For example, spontaneous fermentation under optimized conditions has been shown to yield communities dominated by *Pediococcus* and *Weissella*, reaching pH values between 3.8 and 4.3 and enabling their use as starter cultures for plant-based fermented products [2]. Similarly, the use of selected autochthonous LAB strains, such as *Lactiplantibacillus plantarum* LpAv and *Limosilactobacillus fermentum* Lf2, in sterilized quinoa extract resulted in rapid acidification (pH < 4.5 within 8 h) [32]. In the present study, although a slight decrease in pH was observed (from 6.1 to 4.9), this level of acidification was insufficient to establish a protective environment typically associated with LAB-driven fermentation. This limited acidification, combined with the absence of key metabolic pathways for lactic acid production identified in the metagenomic analysis, likely contributed to the persistence and dominance of non-LAB microorganisms.

LAB commonly associated with rejuvelac, such as *Pediococcus* and *Weissella*, may harbor intrinsic or acquired antibiotic resistance genes (ARG), including determinants related to vancomycin (*van*), tetracycline (*tet*), and macrolide–lincosamide–streptogramin (MLS) resistance [33]. Although these genera are often considered beneficial, their resistome profile deserves careful consideration. *Weissella* species, for instance, have been reported as opportunistic pathogens in rare cases of bacteremia, endocarditis, and abscesses, particularly in immunocompromised individuals, and are known to exhibit intrinsic resistance to clinically relevant antibiotics such as vancomycin [34]. Similarly, *Pediococcus* spp. may carry tetracycline and multidrug resistance genes, which could complicate treatment under specific clinical conditions [35].

In the present study, metagenomic analysis revealed the presence of ARGs within the microbial community, reinforcing concerns regarding the role of spontaneously fermented beverages as potential reservoirs of antimicrobial resistance. Notably, the absence of a LAB-dominated fermentation and the predominance of environmental and opportunistic taxa may further increase this risk, as such communities are often associated with more diverse and mobile resistomes, unlike controlled LAB fermentations, where rapid acidification can limit microbial diversity and reduce the persistence of undesirable organisms. From a One Health perspective [36], the regular consumption of such products as health drinks may contribute to repeated exposure to bacteria carrying clinically relevant ARGs, potentially facilitating their persistence and dissemination within the gut microbiota and the environment [37]. These findings highlight the importance of controlled fermentation strategies not only for microbiological safety but also for mitigating the spread of antimicrobial resistance.

This study underscores the critical need for improved safety standards in artisanal fermented foods. Relying solely on spontaneous fermentation, without proper control measures or the use of defined starter cultures, can lead to unintended microbial compositions that pose significant health risks to consumers. Future research should focus on developing and implementing controlled fermentation strategies for quinoa rejuvelac, such as the use of specific LAB starter cultures, to ensure both the safety and the desired functional properties of the final product. Furthermore, a deeper understanding of the specific metabolic activities and potential virulence of the identified microbial members in this context is warranted. From a practical standpoint, these findings highlight that spontaneous fermentation cannot be assumed to consistently yield a safe or probiotic beverage, particularly when substrates such as quinoa, harboring complex endophytic microbiota, are employed. To mitigate risks and enhance the safety of homemade rejuvelac, several measures can be implemented. The use of defined starter cultures, such as well-characterized lactic acid, can promote rapid acidification and suppress undesirable microorganisms. In parallel, improved hygiene practices, including thorough cleaning and, where feasible, sterilization of containers and utensils, are essential to minimize environmental contamination. The use of safe water sources, preferably boiled or filtered, further reduces the introduction of exogenous microbes. Additionally, controlling fermentation time and temperature, ideally maintaining conditions at 20–25 °C and avoiding prolonged fermentation, can help prevent the overgrowth of opportunistic taxa. Monitoring acidification through simple pH measurements (pH < 4.5) provides an accessible indicator of fermentation success and microbial safety, while pre-treatment of seeds, such as washing, soaking, or mild decontamination, may reduce the load of seed-borne endophytes that could otherwise dominate the process.

## 5. Conclusions

This study provides for the first time a comprehensive microbiological assessment of spontaneously fermented quinoa rejuvelac, revealing critical insights into its microbial composition and associated food safety risks. Our combined 16S rRNA gene sequencing and shotgun metagenomics approach demonstrated a predominant microbial community characterized by *Pseudomonadales*, including genera such as Pseudomonas, Acinetobacter, Enterobacter, and members of *Burkholderiales*. Crucially, the expected beneficial lactic acid bacteria (LAB), vital for safe and stable fermentations, was found to be largely absent or at negligible levels. The recovery of medium- to high-quality metagenome-assembled genomes (MAGs) from *Burkholderiaceae* and *Clostridiales* further substantiated the significant presence of non-beneficial and potentially opportunistic taxa. The findings underscore that the spontaneous fermentation process, particularly when initiated from quinoa seeds with their inherent endophytic microbiota, may inadvertently select environmental and potentially pathogenic microorganisms rather than promoting controlled, safe fermentation. This highlights a significant food safety concern for artisanal rejuvelac production, as the lack of LAB dominance compromises the natural protective mechanisms typically found in fermented foods. Consequently, consumers of such beverages may be exposed to unintended microbial contamination and associated health risks. While quinoa rejuvelac is often perceived as a functional food, its spontaneous preparation method, as investigated herein, presents substantial microbiological hazards. This study strongly encourages the implementation of improved hygiene practices, the use of controlled starter cultures, and robust monitoring strategies to mitigate these risks. Future efforts should focus on developing standardized fermentation protocols to ensure the safety and consistent quality of quinoa-based fermented beverages. In addition, pre-fermentation microbiome analysis of the seeds would be highly valuable to establish a direct link between the initial microbial community and the resulting fermentation outcomes, enabling better prediction and control of the process. Such integrated approaches are essential for protecting consumer health and fully realizing the potential of quinoa as a valuable substrate for functional foods.

## Figures and Tables

**Table 1:** Summary of metagenome-assembled genome (MAG) bins recovered from shotgun metagenomic sequencing of quinoa rejuvelac.

Bin Name	Marker Lineage	# Genomes	# Markers	# Marker Sets	Completeness	Contamination
bin.001	*o__Pseudomonadales*	185	813	308	46.62	0.0
bin.002	*k__Bacteria*	5449	104	58	34.48	0.0
bin.003	*o__Burkholderiales*	107	574	251	86.96	2.59
bin.004	*f__Burkholderiaceae*	91	596	218	95.83	0.75
bin.005	*o__Clostridiales*	50	332	124	98.39	0.6
bin.006	*k__Bacteria*	5449	101	56	19.64	1.79
bin.007	*o__Sphingomonadales*	26	569	293	72.24	0.68
bin.008	*f__Enterobacteriaceae*	223	876	305	54.82	0.38
bin.009	*k__Bacteria*	5449	103	57	22.97	0.0
bin.010	*k__Bacteria*	5449	104	58	32.76	0.0
bin.011	*k__Bacteria*	5449	104	58	10.34	0.0

## Data Availability

All sequence data generated in this study have been deposited in the NCBI BioProject database under accession number PRJNA1435442. BioProject and associated SRA metadata are available at https://www.ncbi.nlm.nih.gov/sra/PRJNA1435442, accessed on 12 March 2026.

## Supplementary Materials

The following supporting information can be downloaded at: https://www.mdpi.com/article/10.3390/life16040556/s1, Table S1: Virulence factors found in genome of strain Bin 4 and its respective genes; Table S2: Virulence factors found in genome of strain Bin 5 and its respective genes; Table S3: Digital DNA–DNA hybridization (dDDH) values between the analyzed genome and closely related species of the genus *Ralstonia*; Table S4: Digital DNA–DNA hybridization (dDDH) values between the analyzed genome and closely related species of the genus *Clostridium*.

## Abbreviations

The following abbreviations are used in this manuscript:

LAB	Lactic Acid Bacteria
MAGs	Metagenome-Assembled Genomes
GTDB-Tk	Genome Taxonomy Database Toolkit
TYGS	Type Strain Genome Server
CARD	Comprehensive Antibiotic Resistance Database
VFDB	Virulence Factor Database
dDDH	Digital DNA–DNA hybridization
